# Labour pain management practices in a teaching tertiary hospital in Botswana: a cross-sectional study

**DOI:** 10.11604/pamj.2026.53.11.49732

**Published:** 2026-01-12

**Authors:** Mamo Woldu Kassa, Alemayehu Ginbo Bedada

**Affiliations:** 1 Department of Anaesthesia and Critical Care, University of Botswana, Gaborone, Botswana,; 2 Department of Surgery, Faculty of Medicine, University of Botswana, Gaborone, Botswana

**Keywords:** Analgesia, barriers, labour, pain, pharmacological, non-pharmacological

## Abstract

**Introduction:**

pain is a universal experience that can lead to distress, anxiety, depression, increased healthcare use, and even adverse outcomes. Labour pain is among the most severe types experienced by women and requires specialized treatment, yet its control remains underutilized in many low- and middle-income countries, including Botswana, where information on current practice is limited.

**Methods:**

a cross-sectional quantitative study on labour pain management practices among healthcare providers was conducted at Princess Marina Hospital from July to August 2025. Using a structured Self-administered questionnaire, data on demographics, analgesia practices, and barriers were collected from eligible providers and analysed using SPSS version-30.

**Results:**

among the 58 labour analgesia care providers invited, 56 providers responded (96.6%). Most were females (69.6%) and midwives (57.1%), aged 31-40 years (46.4%), with over 5 years of experience (57.1%). All obstetricians, and most residents (90.9%) and midwives (84.4%) provided counselling, mainly during labour (83.9%). Pharmacological and non-pharmacological methods were each used by 98.2% of providers. Common analgesics included pethidine (98.2%), paracetamol (50.0%), and morphine (19.6%), while deep breathing (82.1%), massaging (78.6%), and reassurance (69.6%) were common non-pharmacological methods. Only 26.8% of the care providers had formal training in labour pain management. The main barrier to effective analgesia was a lack of training, inadequate equipment and supplies, and fear of fetal distress.

**Conclusion:**

key barriers to labour pain management were inadequate training and a shortage of equipment and supplies. Enhancing capacity building, ensuring resource availability, and developing locally appropriate protocols could significantly improve labour analgesia service and its effective application.

## Introduction

Pain is a universal experience that can cause significant distress, anxiety, depression, increased health care utilization, and even mortality, especially when it persists longer than expected [1,2]. Labour pain, characterized by unique physiological changes and emotional components, requires special management [3,4]. During the first stage of labour, pain is visceral in nature, arising from uterine contraction and cervical dilatation. In the second stage, it becomes somatic, resulting from distension of the vagina, perineum, and pelvic floor [4]. Its intensity varies among parturient mothers; those who are afraid of birth often experience more severe pain than those with a “no pain, no gain” attitude and adequate support [3]. Factors influencing labour pain include prior childbirth experience, personal expectations, quality and level of support from care providers, involvement in decision-making, parity, culture and ethnic background, and individual coping ability [4-6].

Parturient mothers receive care from various healthcare providers, including obstetricians, residents, medical officers, and midwives [1]. Effective labour pain management depends on the provider´s experience, values, and interest, as well as the facility resources, stage of labour, maternal involvement, and the medical condition of both mother and fetus [1,2,7,8].

Effective labour pain management combines non-pharmacological interventions that help mothers cope with pain and pharmacological methods that provide pain relief [4,7,9]. Parturient mothers should be free to choose their preferred pain management approach and be well informed about the available options for themselves and their babies [4]. While effective labour analgesia does not increase the rate of caesarean delivery, it may prolong the second stage of labour and slightly increase the rate of instrumental vaginal delivery [10,11].

Various pharmacological methods are used for labour analgesia, either alone or in combination, including non-steroidal anti-inflammatory drugs (e.g., diclofenac), opioids (e.g., pethidine, morphine, fentanyl), inhaled nitrous oxide, local anaesthetics for nerve blocks, and intrathecal/epidural injections of local anaesthetics and/or opioids. These methods differ in maternal satisfaction, effects on labour progress, rates of caesarean section and instrumental deliveries, and potential side effects for the mother and fetus [2,4,8,10,12-15]. Barriers to effective pharmaceutical pain management include high workload, inadequate skills and knowledge, limited personnel, fear of adverse effects of analgesics on the mother and fetus, and shortage of medication and infrastructure, often leading to maternal dissatisfaction [16-18].

Non-pharmacological interventions such as immersion in water, relaxation techniques (e.g., music), acupuncture, and massage provide effective pain relief and enhance maternal satisfaction [4]. However, their application is often limited by factors such as patients´ beliefs, lack of time, insufficient skills and knowledge, and inadequate staffing [16,17,19].

Labour pain management in low- and medium-income countries is influenced by economic constraints, technical challenges, limited knowledge and experience, medicolegal issues, maternal and fetal considerations, costs, and patient expectations [7,18,20]. Services are generally underdeveloped, poorly established, and sparsely documented in low- and middle-income countries [7,9,18,20-22]. To date, there is no published information on labour management practices in Botswana. This study aims to assess the practice of labour pain management among providers at Princess Marina Hospital (PMH), examining provider characteristics, types of analgesia used, and barriers to practice. Findings may help clinicians to appreciate the gaps in practice and areas for improvement, guide policymakers in resource allocation, and inform context-specific labour pain care in similar settings.

## Methods

**Study design:** we conducted an institution-based, cross-sectional quantitative study on labour pain management practices among labour analgesia providers at PMH.

**Study setting and population:** located in Gaborone, the capital of Botswana, PMH is the country´s largest tertiary public teaching hospital and is affiliated with the University of Botswana. It serves approximately one million people in the southern region of the country. The study was conducted between 1^st^ July and 31^st^ August 2025. A structured, self-administered questionnaire was distributed to all obstetricians, obstetrics-gynaecology residents, medical officers, and midwives involved in maternal care at PMH. All labour analgesia providers who met the inclusion criteria were invited to participate in the study. Notably, during the study period at PMH, there was no formal or documented labour pain management protocol, anaesthesiologists did not provide labour pain analgesia, and epidural analgesia services were unavailable.

**Variables:** variables of interest included age, gender, professional positions, years of experience, counselling, analgesia methods and agents, and their potential side effects, availability of training, equipment, and supplies, and staff shortages. Gender and professional positions were used as independent variables, while the use of analgesia serves as the dependent variable.

### Data sources

**Data collection tool:** data were collected using a structured, self-administered questionnaire. The questionnaire was adopted from similar research that examined epidemiology, care providers´ experiences, analgesia practices, and barriers to implementation, and was contextually modified for our setting. The questionnaire variables were tested for clarity with an obstetrician, a resident, a medical officer, and a midwife, and no revisions were required (Annex 1).

**Data collection:** the data was collected by the study principal investigator (PI) and the form included: section-1: demographics - included gender, age group, professional qualification, and years of experience. Section-2: clinical practices - covered counselling on labour pain, timing of counselling, and the methods and types of pain relief utilized, and section 3: institution factors - explored aspects such as the current pain management practices, training or involvement in related discussions, types of pain management practiced, perceived barriers to effective labour pain management, and shortages in staff, resources, and equipment. After capturing the variables of interest in an Excel sheet on a password-protected computer, the data collection form was securely stored in a locked cabinet in the PI´s office.

**Sample size:** a purposive sampling method was used, including all clinicians involved in maternal care at PMH during the study period. This approach allowed the inclusion of participants with relevant experience and knowledge, ensuring rich and targeted information for the study. However, it may introduce selection bias and limit the generalizability of the findings to other settings. According to unpublished PMH labour ward documents, the average number of labour analgesia providers over the past five years at PMH was 55.

**Data analysis:** providers´ characteristics - reported by position and age group and described using proportions. Practice of labour pain management - counselling and the use of pharmacological and non-pharmacological methods by analgesia providers are presented in proportions. Types of analgesia used - classified into opioids and non opioids, further subclassified into specific agents, and reported in absolute numbers and proportions. Barriers to practice - common barriers, including inadequate training, lack of equipment and supplies, and fear of fetal distress, are described using proportions. A Chi-squared test was performed to assess the association between gender (male vs. female) and position (doctors vs. nurses) with the provision of labour analgesia, with a p-value of <0.05 considered statistically significant. Data analysis was performed using IBM SPSS Statistics, Version 30.

**Ethical consideration:** ethical approval for the study was obtained from the PMH Institutional Review Board (ref. HPRD: 6/14/1, dated 5^th^ May 2025). Written informed consent was obtained from all providers after a detailed explanation of the study purpose.

## Results

**Figure 1:**
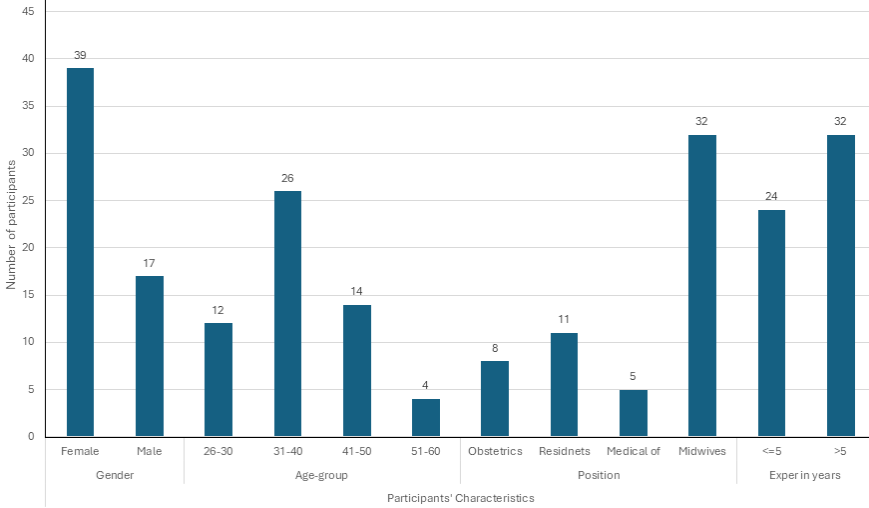
labour analgesia providers' characteristics (n=56) at the Princess Marina Hospital from July to August 2025

**Providers’ characteristics:** during the study period, PMH had nine obstetricians, 12 gynecology-obstetrics residents, five medical officers, and 32 midwives providing care to labouring mothers. Except for one obstetrician and one resident, 56 out of 58 providers completed the survey, yielding a response rate of 96.6%. Females comprised 39 (69.6%) of the respondents, while males accounted for 17 (30.4%). Among the providers, midwives represented the largest group, 32 (57.1%), followed by residents 11 (19.6%), obstetricians eight (14.3%), and medical officers five (8.9%). Most providers were aged 31-40 years, 26 (46.4%), and the majority had more than five years of experience, 32 (57.1%) (Figure 1).

### Labour analgesia provision

**Counselling about labour pain before, during, and after delivery:** all obstetricians and residents provided counselling on labour pain, while 27 midwives (84.4%), and four medical officers (80.0%) did so. Counselling was offered before labour, by 39 providers (69.6%), during labour by 47 (83.9%), and after delivery by 28 (50.0%). Notably, seven providers (12.5%) offered no counselling at any stage of labour. Doctor providers offered more comprehensive counselling before, during, and after delivery compared to nurse providers, 91.7% vs. 84.4%, respectively, p=0.686.

**Pharmacological and non-pharmacological labour analgesia methods:** overall, 55 care providers (98.2%) used pharmacological methods, while one midwife used only non-pharmacological methods (1.8%). During the first stage of labour, 46 care providers (82.1%) used both pharmacologic and non-pharmacologic methods, while seven (12.5%) used only pharmacologic methods, two (3.6%) used only non-pharmacological methods, and one (1.8%) used neither. In the second stage, 15 providers (26.8%) used both methods, three (5.4%) used only pharmacological methods, 24 (42.9%) used non-pharmacological methods, and 14 (25.0%) used neither approach.

**Figure 2:**
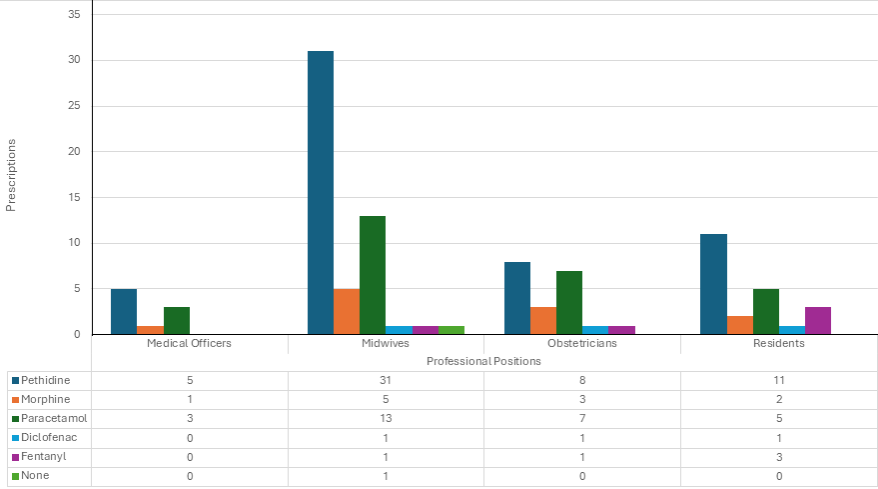
pharmacological methods prescribed by the labour analgesia providers at the Princess Marina Hospital from July to August 2025

Pharmacological methods were reported 102 times, with opioids used in 71 instances (69.6%) and non-opioids in 31 instances (30.4%). Among the pharmacological analgesia, pethidine was the most commonly used by 55 providers (98.2%), followed by paracetamol 28 (50.0%), morphine 11 (19.6%), fentanyl five (8.9%), and diclofenac three (5.4%). Doctor providers offered more pharmacological methods of labour pain analgesia compared to nurse providers, 100.0% vs. 96.9%, respectively, p=1.000 (Figure 2). The majority, 55 of the providers (98.2%), reported using at least one non-pharmacological method. The commonest methods of non-pharmacological methods included 46 deep breathing (82.1%), 44 massaging (78.6%), 39 reassurances (69.6%), and 28 encouraged movement (50.0%) (Table 1).

**Table 1:** non-pharmacological methods prescribed by labour analgesia providers at the Princess Marina Hospital from July to August 2025

	Medical officer	Midwives	Obstetricians	Residents	Total
Deep breathing	4 (80.0%)	26 (81.3%)	8 (100.0%)	8 (72.7%)	46 (82.1%)
Massaging	4 (80.0%)	24 (75.0%)	8 (100.0%)	8 (72.7%)	44 (78.6%)
Reassurance	3 (60.0%)	22 (68.8%)	7 (87.5%)	7 (63.6%)	39 (69.6%)
Moving around	3 (60.0%)	14 (43.8%)	6 (75.0%)	5 (45.5%)	28 (50.0%)
Exercise	3 (60.0%)	15 (46.9%)	2 (25.0%)	3 (27.3%)	23 (41.1%)
Position change	1 (20.0%)	12 (37.5%)	3 (37.5%)	3 (27.3%)	19 (33.9%)
Psychological support	2 (40.0%)	9 (28.1%)	1 (12.5%)	2 (18.2%)	14 (25.0%)
Advise to bear	0 (0.0%)	7 (21.9%)	4 (50.0%)	3 (27.3%)	14 (25.0%)
Heat/ice	1 (20.0%)	6 (18.8%)	1 (12.5%)	2 (18.2%)	10 (17.9%)
None	0 (0.0%)	0 (0.0%)	0 (0.0%)	1 (9.1%)	1 (1.8%)

**Health system factors:** fifteen providers (26.8%) indicated having received training in labour pain management. Fifty-two providers (92.9%) had engaged in discussions about labour pain management, with nine (16.1%) participating always, 21 (37.5%) often, and 22 (39.3%) involved sometimes. Regarding the provision of labour pain management services, providers believed it was offered always in 17.9% of cases, often in 51.8%, sometimes in 25.0%, and never in 5.4%.

The top three barriers for the provision of labour pain management services were lack of training in 66.1%, lack of equipment and supplies in 58.9%, and fear of fetal distress in 57.1% (Table 2). Female providers reported providing labour analgesia more frequently than male providers, 94.9% vs. 82.4%, respectively, though the difference was not statistically significant, p=0.158. Doctors (obstetricians, residents, and medical officers) were more likely to provide labour analgesia than nurses, 100.0% vs. 84.4%, p=0.064, respectively.

**Table 2:** barriers for the provision of labour pain management services at the Princess Marina Hospital from July to August 2025

	MOs*	Midwives	Obstetricians	Residents	Total
Lack of training	3 (60.0%)	18 (56.3%)	7 (87.5%)	9 (81.8%)	37 (66.1%)
Lack of equipment and supplies	5 (100.0%)	12 (37.5%)	7 (87.5%)	9 (81.8%)	33 (58.9%)
Fear of fetal distress	1 (20.0%)	20 (62.5%)	4 (50.0%)	7 (63.6%)	32 (57.1%)
Shortage of trained staff	1 (20.0%)	13 (40.6%)	6 (75.0%)	10 (90.9%)	30 (53.6%)
Lack of monitors	3 (60.0%)	11 (34.4%)	6 (75.0%)	9 (81.8%)	29 (51.8%)
Fear of maternal respiratory depression	1 (20.0%)	4 (12.5%)	0 (0.0%)	2 (18.2%)	7 (12.5%)
Increased duration of labour	0 (0.0%)	0 (0.0%)	3 (37.5%)	2 (18.2%)	5 (8.9%)
No maternal request	0 (0.0%)	2 (6.3%)	0 (0.0%)	2 (18.2%)	4 (7.1%)
Fear of maternal fever and infection	0 (0.0%)	1 (3.1%)	0 (0.0%)	1 (9.1%)	2 (3.6%)
Increased risk of CS^β^	0 (0.0%)	1 (3.1%)	1 (12.5%)	0 (0.0%)	2 (3.6%)
Concern about cost	1 (20.0%)	0 (0.0%)	0 (0.0%)	1 (9.1%)	2 (3.6%)

* MOs: medical officers; CS^β^: caesarean section

Among the providers, the shortage of medical officers, 42 (75.0%), was identified as the primary staffing challenge, followed by the shortage of nurses, 41 (73.2%), and specialists, 38 (67.9%). The majority of providers, 91.1%, indicated that establishing labour analgesia services at PMH is necessary, and 76.8% believed it is feasible. Notably, 80.4% supported the inclusion of epidural labour analgesia. All obstetricians, residents, and medical officers expressed the need for the establishment of labour analgesia services, compared to 84.4% of midwives. At Princess Marina Hospital, the lack of anaesthesiologists, 67.9%, was cited as the main barrier to providing safe epidural labour analgesia, followed by lack of knowledge, 66.1%, and the absence of appropriate monitoring equipment, 66.1%. Most providers, 38 (67.9%), agreed that labour analgesia services should be led by a specialist.

## Discussion

In this study, we explored labour pain management practices among obstetricians, residents, medical doctors, and midwife nurses currently practicing in the labour ward. Labour pain counselling before the onset of labour was provided by 69.6% of care providers. Pethidine (98.2%) was the most commonly used pharmacological method of analgesia, while deep breathing (82.1%) was the main non-pharmacological method. The most frequently reported barriers to labour analgesia services were lack of training (66.1%), lack of appropriate equipment and supplies (58.9%), and concern about fetal distress (57.1%). The primary barrier to the use of epidural labour analgesia was the limited number of anaesthesiologists.

**Providers’ characteristics:** in our study, the response rate was 96.6%, which is comparable to previous reports ranging from 74.0% to 99.7% [7,23-25]. Female care providers dominated, accounting for 69.6%, consistent with a study from Nigeria where females constituted 67.4% of providers. However, another study from Nigeria and two Ethiopian studies reported male predominance, ranging from 54.5% to 89.4% [23,24,26]. The most common age group among our care providers was 41-50 years, 46.4%, which is similar to findings by Lawani *et al*. from Nigeria, 52.3%, in the same [23], whereas Terfasa *et al*. [24] reported a younger age distribution, with 70.4% of providers aged 20-29 years in Ethiopia. In our study, midwives were the main providers of labour analgesia, 57.1%, aligning with Ethiopian studies where 51.7%-57.8% of providers were midwives [24,26]. In contrast, a study from Nigeria reported that medical doctors were the main providers, 49.5% [25]. The majority of our labour analgesia providers, 57.1%, had more than 5 years of work experience, similar to a Nigerian study reporting 58.9% with over five years´ experience [23]. Ethiopian studies found that most providers, 66.6%-75.6%, had less than five years´ experience [24,26]. The differences or similarities in gender and age group among different countries could be due to recruitment to fill gaps based on the availability of the providers and need of the country, economic development and employment stability, gender roles and cultural norms, migration and brain drain, professional advancement opportunities, and societal and family factors.

**Labour analgesia provision:** though some labour pain care providers, 3.2%-50%, believe labour pain is a natural process and necessary for birth, and it does not need routine pharmacological pain relief, but many more, 82.1%-94.8%, indicated the need for providing labour analgesia [7,9,24-27]. Ouma *et al*. from Kenya reported a 17.1% rate of uncertainty about the need for labour analgesia among their providers [7]. Our providers indicated the need for labour analgesia in 91.1% of the cases. Among obstetricians, 13.3% offered labour analgesia routinely, 29.1% sometimes, and 6.6% on patients' request [7], while 100.0% of our obstetricians indicated providing labour analgesia routinely. The variation in labour analgesia among different health institutions could be due to knowledge and awareness difference, attitude and belief about pain and analgesia, availability and accessibility of medications, training and professional experience, patient and fetus-related factors, and fear of adverse effects and local regulations.

More labour analgesia is provided by females and medium and highly qualified care providers [26]; this is consistence with our findings, where our female than male and doctor than nurse providers have more prescriptions of labour analgesia. This could be due to the personal experience with childbirth, which enhances empathy towards women in labour. Female clinicians tend to adopt a more patient-centred approach and feel more comfortable discussing sensitive topics like labour pain and maternal comfort. Female patients in labour might feel more comfortable expressing pain and requesting relief from female clinicians, which can be translated to the frequent use of labour analgesia by female providers than males.

The level of knowledge among the care providers about labour analgesia varies from 58.0% to 100.0% [22,26]. In our study, the majority of the care providers provide counselling about labour pain management mainly during labour, 83.9%. The rate of usage of combined pharmacological and non-pharmacological methods varies from 84.2% to 88.9% [7,24], only pharmacological methods ranging from 11.1% to 44.5% [7,24], and only non-pharmacological methods in 54.7% of the cases [7]. The usage also varies; some use routinely, others use sometimes or upon maternal request at various rates [9,24]. In our study, the rate of usage of both methods of labour analgesia was 98.2%. Our providers did not use pharmacological methods in isolation, but one of our providers (1.8%) used only a non-pharmacological method. Various types of labour analgesia were used in different studies; non-steroidal anti-inflammatory analgesia was used from 1.2% to 70.9% and it included ibuprofen and diclofenac [7,9,24,26]. Paracetamol was used from 4.6% to 18.3% [23,27], diclofenac 3.4% to 16.8%, and hyoscine in 58.6% [27]. In our study, paracetamol and diclofenac were used in 28/56 (50.0%) and 3/56 (5.4%) of the cases, respectively. Opioids such as morphine, codeine, pethidine, and tramadol were used in 7.3% to 48.7% of the cases [7,23,24,26]. Pethidine was used in 6.9% to 17.1% [24,27] and tramadol in 88.9% of the cases [7]. Our labour analgesia providers used pethidine in 55/56 (98.2%), morphine in 11/56 (19.6%), and fentanyl in 5/56 (8.9%) of the cases.

Epidural labour analgesia rates were reported in 0.6% to 24.9% of the cases [7,9,23,24,26,28], while cervical/local anaesthesia was reported in 1.8% of the cases [26]. Some researchers indicated that 13.6% of the labour analgesia providers were not aware of the inhalational anaesthesia method [9,26]. Although epidural analgesia remains the most effective method for labour pain management, its utilization is constrained by systemic barriers in our setting, and no epidural or inhalational labour analgesia was used by our labour analgesia providers. In low- and middle-income countries, the absence or low utilization of epidural, nerve block, or inhalational labour analgesia is multifactorial, including knowledge gaps, staff shortages, resource limitations, cultural beliefs, and weak institutional supports.

Various non-pharmacological methods are in use among labour analgesia providers to support labouring mothers. These methods include comforting, counselling, massage, breathing exercise, encouraging the presence of a companion, taking a bath, ambulating, and reassurances [4,7,9,24,26]. The rate of use of non-pharmacological methods of labour pain management ranges from 15.4% to 93.8% [7,23,24,26,27]. Massaging was the most commonly used non-pharmacological method in 80.3% to 93.8% [7,26], followed by position changing in 11.6% to 81.3% [7,24,26], allowing the labouring mother to move around in 81.3% to 93.7% [7,24,26], reassurance in 79.7%, and deep breathing in 78.4% of the cases [7]. Our providers indicated that they used non-pharmacological methods in 98.2% of the cases. The three most commonly used non-pharmacological methods in our study were deep breathing in 82.1%, massaging in 78.6%, and reassuring in 69.6% of the cases.

**Factors influencing labour pain management:** the majority of labour analgesia providers have a positive attitude regarding labour pain management [9]. Labour analgesia management is influenced by a complex interplay of maternal, fetal, clinic, and institutional factors. The knowledge of the labour analgesia providers about the effect of pharmacological agents on the progress of labour, maternal health and fetal distress, rate of instrument delivery, and caesarean section is critical [9,24,27]. Lack of equipment and supplies was indicated in 58.9% of our labour analgesia providers, which is in agreement with previous reports that range from 42.7% to 58.1% [7,24]. Fear of fetal distress was indicated in 57.1% among our labour analgesia providers; previous studies reported 31.7% to 69.5% for the same [7,24]. Fear of increased rate of caesarean section was reported in 14.6% [24], our providers feared in 3.6% of the cases. Delay in progress of labour was reported from 63.6% to 66.6% [7,24], which was reported in 8.9% of our cases. Shortage of trained staff was indicated from 55.6% to 85.7% [7,24], our labour analgesia providers indicated the shortage in 53.6% of the cases. Some researchers emphasized the introduction of labour pain management protocol and regular staff training to improve labour analgesia services [7]. Lack of training was indicated in 66.1% of cases among our care providers, while others reported from 55.6% to 85.7% of the cases [7,24].

**Strengths and limitations:** although we conducted the study in the largest public hospital in the country, our single hospital-based study and medium sample size limit its generalizability. To mitigate this effect, we included all labour analgesia provided in the hospital, with a response rate of 96.6%. To the best of our knowledge, this is the first study conducted in Botswana, and it identified unmet local labour analgesia needs. It will help clinicians to recognize the gaps in practice and areas for improvement. It supplies local evidence for policy formulation and resource allocation, and it provides context-specific care for patients in similar settings. It also provides a comparative perspective and diversity of clinical realities for international readers.

## Conclusion

All our providers provided labour analgesia. The majority were aged between 31 and 40 years, and most had over five years of professional experience. Counselling on labour analgesia was provided by 69.6% of the providers. Both pharmacological and non-pharmacological methods were used by 98.1% of the providers. Pethidine was the most commonly used pharmacological agent, while deep breathing exercise was the most frequently applied non-pharmacological technique. Pharmacological methods were most commonly used during the first stage of labour, whereas non-pharmacological techniques predominated during the second stage. The main barriers to effective labour pain management included lack of training, shortage of equipment and supplies, and limited availability of doctors, particularly anaesthesiologists, whose scarcity was the major obstacle to providing epidural labour analgesia. We recommend strengthening capacity building and ensuring the availability of the required drugs, equipment, and supplies, which could significantly improve labour analgesia services. Furthermore, the development of contextually appropriate labour analgesia management protocols is crucial to ensure safe, effective, and dignified pain relief for labouring mothers.

### What is known about this topic

- Labour analgesia is a vital component of labouring mothers; however, it remains underutilized and poorly documented in many sub-Saharan African countries;
- The provision of labour analgesia is often constrained by shortages of the required drugs, equipment, and trained personnel, and institutional and cultural barriers;
- Providers´ knowledge, attitudes, and practices regarding labour analgesia are key determinants of the quality and delivery of this critical healthcare service.

### What this study adds

- We identified context-specific evidence on labour analgesia practices and staff perspectives at Princess Marina Hospital in Botswana;
- We identified critical barriers to labour analgesia, including gaps in personnel training, limited resources, and the absence of standardized protocol;
- We established baseline data to inform the development of labour analgesia guidelines and protocols, and to guide policymakers in ensuring adequate staffing and resources.

## Annex

**Annex 1**: supplementary materials (PDF 195KB)
